# Is early full weight bearing safe following locking plate ORIF of distal fibula fractures?

**DOI:** 10.1186/s12891-021-04009-x

**Published:** 2021-02-09

**Authors:** Michael Zyskowski, Markus Wurm, Frederik Greve, Sebastian Pesch, Francesca von Matthey, Patrick Pflüger, Moritz Crönlein, Peter Biberthaler, Chlodwig Kirchhoff

**Affiliations:** Klinikum rechts der Isar, Technische Universität München, Klinik und Poliklinik für Unfallchirurgie, Ismaninger Str. 22, 81675 Munich, Germany

## Abstract

**Background:**

In the modern western world appraisal of economical points such as treatment and disability after trauma present a financial burden. In this context open reduction internal fixation techniques allowing for early full weight bearing might not only improve the clinical outcome but also shorten the period of disability in working life. The aim of the study was to analyze whether ORIF of ankle fractures using either a standard semitubular plate or a new polyaxial locking plate system result in a better clinical outcome.

**Methods:**

In this prospective study, all patients with distal fibula fractures (AO 44 B1.1, B1.2, B1.3), with indication for surgery were included. Patients were randomized to either the DePuy Synthes® one-third semitubular plate (Group I) or NEWCLIP TECHNICS, Active Ankle® polyaxial locking plate (Group II). Primary outcome parameter was function of the ankle joint, assessed by the Olerud and Molander ankle score, Foot and Ankle outcome score and Karlsson and Peterson Scoring System for Ankle function. Secondary outcome parameter were postoperative complications. Superficial wound infection, delayed wound healing, mechanically prominent implant, skin irritations were considered as minor and deep wound infection, material loosening, loss of reduction were regarded as major complications requiring revision surgery. Clinical and radiological follow-up were performed 6 and 12 weeks, 6 months and 1 year postoperatively.

**Results:**

Fifty-two patients (31 W/21 M) with a mean age of 43 yrs. (range 22–64 yrs.) were enrolled. Seven patients (13.5%) were excluded, so that 45 patients were available for follow up. Twenty-five patients were treated with DePuy Synthes® one-third semitubular plate (55.6%; group I) while 20 patients received an anatomically preformed polyaxial locking plate (44.4%, group II). Four minor complications occurred in Group I (16%) compared to two minor complications in group II (10%). Significant better clinical results regarding OMAS (*p* < 0.02, < 0.04), KPSS (*p* < 0.04) and FAOS (*p* < 0.02, < 0.03) were observed 6 and 12 weeks after surgery in group II.

**Conclusions:**

The results of the presented study demonstrate a significant better clinical functional outcome in the early postoperative follow-up in patients treated with a polyaxial locking plate. Furthermore, our data show that ORIF using polyaxial locking plates in combination with an early postoperative weight bearing presents a safe, stable treatment option for ankle fractures so that patients benefit especially in the early stages of recovery.

**Trial registration:**

Registered 20 April 2020, retrospectively on ClinicalTrails.gov (NCT04370561).

## Background

Ankle fractures (FX) are common injuries, accounting for 9% of all fractures of the human skeleton [1]. In the current literature multiple studies compare conservative and surgical treatment in malleolar FX [2–4]. In this context open reduction and internal fixation (ORIF) presents the standard of care for displaced ankle FX in adults [4–6]. Several techniques for internal ankle fixation are commonly used, ranging from lag screw fixation to plate osteosynthesis with non-locking to locking screw systems up to biodegradable types [7–10]. Nevertheless operative treatment is associated with typical complications such as non- or malunion, post-traumatic arthritis and especially soft tissue problems ranging from delayed wound healing to deep infection and severe soft tissue defects [11–15].

Besides fracture healing especially postoperative joint rehabilitation determines functional outcome and thereby return to sports, work and normal daily activities. Most patients suffering from ankle FX are in the middle of their individual working life (5th decade) [16–18] and usually demand early return to daily activities. Therefore, the main goal of any post-operative therapy is to reduce time of recovery to a minimum and to achieve full physical performance as early as possible. In the past a few clinical trials revealed that early weightbearing and functional treatment avoiding a plaster cast may shorten the immobilization period but may provoke a loss of reduction depending on morphology, initial stability of the FX as well as on patient’s age and comorbidities [19–24].

In this context biomechanical cadaver specimen studies suggest an advantage of locking plates in osteoporotic bone [7, 9, 25–27]. Moreover, anatomically preformed implants with polyaxial locking mechanisms revealed promising results in the treatment of complex FX including distal femur [28], tibia [29], radius [30], clavicle [31] and the radial head [32]. First retrospective clinical studies revealed a benefit of locking plates in osteoporotic patients [26, 33] but also persisting high complication rates [12, 13, 15]. Nevertheless locking plate systems are still under economical discussion because of high implant costs [13, 15, 26, 34, 35].

To the best of our knowledge there is still no prospective randomized trial comparing non-locking and locking plate osteosynthesis of distal fibula FX. Therefore, the aim of this study was to analyze whether the use of one system leads to better clinical outcome and or a reduction of complications.

## Methods

### Patients

Institutional Review Board approval was obtained prior to this study (IRB approval No: 429/15, Ethical Committee of Technical University Munich). The randomized controlled trials registration was assigned (NCT04370561). All patients who underwent surgery at our academic level-one trauma center between 01/2016 and 01/2018 for a distal fibula FX type 44 B1.1, B1.2, B1.3 according to AO classification system (age 18–65 yrs) were prospectively enrolled. Written informed consent was obtained for each patient. Patients were randomly assigned (Randlist®, DatInf GmbH, Tübingen, Germany) to either group I – non-locking (DePuy Synthes® one-third semitubular plate; DePuy Synthes®, Umkirch, Germany) or group II – locking (NEWCLIP TECHNICS® Active ankle system 2,8/3,5 mm, Pa De La Lande Saint Martin, France).

Exclusion criteria were pregnancy, mental disorders, open FX as well as comprehensive legal support. Patients with pathological, osteoporotic and open FX were excluded as well.

### Surgical technique and postoperative protocol

All patients were operated by lower extremity expert trauma surgeons undergoing general anesthesia. Thirty minutes prior to surgery a single prophylactic dose of 1.5 mg cephalosporin was administered. For surgery patients were placed in a supine position on a radiolucent table with a pillow under the ipsilateral to the injured ankle gluteal region. Under tourniquet control (250 mmHg) a standard lateral approach to the distal fibula was performed according to AO recommendations. In general the aim was to place the plate in a lateral position. However, due to the plate design the anatomically preformed polyaxial locking plate tends to a slight anterolateral position. In group I-patients ORIF was performed using a 3.5 mm lag screw and semi-tubular neutralization plate as well as 3.5 mm cortical screws according to AO recommendations [36].

In contrast in group II - patients a reposition clamp allowed for a temporary fixation of the fracture, so a lag screw was not needed. In the following the anatomically preformed plate was inserted so that the distal polyaxial 2.8 mm locking holes were positioned under fluoroscopy control in the epiphyseal region distal to the Fx. A 3.5 mm cortical screw in the oblong hole secured the plate’s position. Diaphyseal 3.5 mm screws were used as standardized locking screws. This surgical technology allows for a locking of the screws along with the plate.

In all patients physiotherapy was initiated on the second postoperative day. Preoperative ASA Physical Status Classification System was collected from the anesthesia documentation [37].

Group I – patients were treated following rehabilitation protocols (see Table 1) including partial weight bearing restricted to 20 kg for 6 weeks on crutches using a medical walking boot and allowing for pain-adapted motion out of the walking boot without limitations according to the recommendations of the German Society for Orthopedics and Trauma (DGOU) [38, 39]. After the initial 6 weeks patients were allowed to increase the weight bearing load with the goal to achieve full weight bearing 8 to 10 weeks after surgery.

**Table 1:** Rehabilitation protocol for both study groups

Rehabilitation protocol	Week 1–6 after surgery	Week 7–12 after surgery
**DePuy Synthes® one-third semitubular plate**	partial weight bearing (20 kg)	Increasing weight bearing, goal: full weight bearing in 10 weeks
	walking boot	train away the walking boot
	crutches	crutches till full weight bearing
	pain-adapted motion, no limitation	pain-adapted motion, no limitation
	**Week 1–3 after surgery**	**Week 4–12 after surgery**
**Newclip Technics® Active ankle system**	partial weight bearing (20 kg)	pain adapted full weight bearing
	walking boot	train away walking boot switch to ankle brace
	crutches	no crutches
	pain-adapted motion, no limitation	pain-adapted motion, no limitation

In group II partial weight bearing was restricted to 20 kg for only 3 weeks using a medical walking boot and crutches as well as pain-adapted motion out of the walking boot without limitations with the goal to achieve proper wound healing. After 3 weeks full weight bearing was allowed.

Both groups received daily subcutaneous thrombosis prophylaxis with low-molecular-weight heparin until full weight-bearing was achieved.

### Follow-up evaluation

The first follow-up exam was set 6 weeks after surgery. Additional follow-ups were performed 3, 6 and 12 months postoperatively. The follow-up examinations were performed by independent investigators not involved in patients’ initial surgical treatment (SP, FG, MW) at the outpatient clinic of our level-one university trauma center.

For assessment of pain, the visual analogue scale (VAS) [40], ranging from 0 “no pain” to 10 “worst imaginable pain” was used. Range of motion (ROM) and ligament stability were registered during standardized clinical follow up examination. For the assessment of the lower extremity and ankle function the Olerud and Molander ankle score (OMAS) [41], Foot and Ankle Outcome Score (FAOS) [42] and the Karlsson and Peterson Scoring System for Ankle function (KPSS) [43] were comprised. Postoperative X-rays were evaluated with special respect to bony healing and secondary loss of reduction.

In addition, sensomotoric disorders and postoperative complications were recorded. Complications such as superficial wound infections, delayed union were considered as minor with the possibility of conservative treatment whereas major complications were regarded if operative revision was needed (e.g. secondary loss of reduction, non-union, severe wound infections etc.).

### Statistics

Statistical analyses were performed using SigmaStat (version 3.5; Systat Software, San Jose, CA, USA). The scores at certain follow-up time points were compared using an independent t-test after a normality check was passed and equal variances were assured. Normal distributed data with unequal variances were compared using the Welch’s t-test. Arbitrarily data was tested with the Mann-Whitney U test. The significance level was set at *p* = 0.05.

## Results

### Epidemiological data

Fifty-two patients were enrolled in the presented study. The mean age was 43 years (range 22–64 years). Regarding the fracture side 27 right (51%) compared to 25 left ankles (49%) were affected. Three patients were lost to follow-up for unknown reasons. Another patient was excluded due to acute leukemia with the need for oncologic treatment just before the first follow-up. After intraoperative syndesmotic stress test one patient received a syndesmotic screw and was therefore excluded. Two patients attended only the first follow up and never returned for further postoperative clinical and radiological control. Finally 45 of 52 patients (86.5%) were available for all follow up examinations, and thus enrolled had a mean age of 42 years (range 22–64 years) at the time of injury with no statistical difference between both groups.

Twenty-five patients were assigned to group I (55.6%) while 20 patients (44.4%) formed group II.

Most common injury was ankle spraining along with supination and external rotation trauma according to the Lauge and Hansen classification [44, 45]. The majority of these injuries happened during recreational time (*n* = 41, 91.1%), only 4 (8.9%) during working hours.

Regarding gender distribution 18 male patients (40%) compared to 27 female patients (60%) were included. The interval between trauma and surgery accounted for an average of 8 days (3–13 days): Group I - 8 days (4–13 days) vs. Group II - 7 days (3–12 days) (see Table 2).

**Table 2:** Interval between trauma and surgery

Interval between trauma and surgery	days
general	⌀8 days
DePuy Synthes® one-third semitubular plate	⌀8 days
Newclip Technics® Active ankle system	⌀7 days

For the fracture side 24 right (53.3%) compared to 21 left (46,7%) ankles were fractured.

The body mass index (BMI) for both groups was almost equal with a BMI of 24 in Group I and of 25 for Group II (see Table 3). The ASA-Score showed no significant differences between the two groups.

**Table 3:** Patients’ demographics and injury characteristics of both groups

DePuy Synthes® one-third semitubular plate	Characteristics	NEWCLIP Tecnics® Active ankle system
⌀43 +/−10	Mean age (years)	⌀38.5 +/−11
9:16	Sex (male/ female)	9:11
15:10	Side (right/left)	9:11
⌀24	Mean BMI	⌀25
	**ASA Scoring**	
15	ASA I	15
8	ASA II	5
1	ASA III	0

### Clinical outcome

In the clinical follow-up, group I presented with twice as many (*n* = 4, 16%) minor complications compared to group II (*n* = 2, 10%).

Minor complications in group I included two cases of swelling and redness of the wound (8%), one deep vein thrombosis (4%) and one superficial infection (4%) treated by intravenous antibiotics for 1 week without the need of implant removal.

In group II two patients (10%) suffered from skin irritations such as mild pain around the suture and scar respectively along with increased skin tension, here the implanted plate was palpable. Also in these two patients no implant removal was necessary but the skin irritations were treated by untightening of the walking boot.

Also for major complications group I presented twice as many (*n* = 2, 8%) compared to group II (*n* = 1, 5%) (see Table 4). In detail, group I showed two deep wound infections with the need of intraoperative debridement and intravenous antibiotics administration for 10 days. In group II, one patient (5%) suffered from a deep wound infection affecting the implant resulting in screw loosening. Here an early implant removal, debridement, and conversion to a conservative treatment regimen in terms of cast immobilization and intravenous antibiotic administration was considered.

**Table 4:** Overview of complications following fracture treatment for both groups

Plating System		minor complications	major complications
**DePuy Synthes®**	**Total**	**4 (16%)**	**2 (8%)**
	swelling and redness	2 (8%)	
	deep vein thrombosis	1 (4%)	
	superficial infection	1 (4%)	
	deep wound infection		2 (8%)
**Newclip Technics® Active ankle system**	**Total**	**2 (10%)**	**1 (5%)**
	skin irritations by the implant	2 (10%)	
	deep wound infection		1 (5%)

**Fig. 1:**
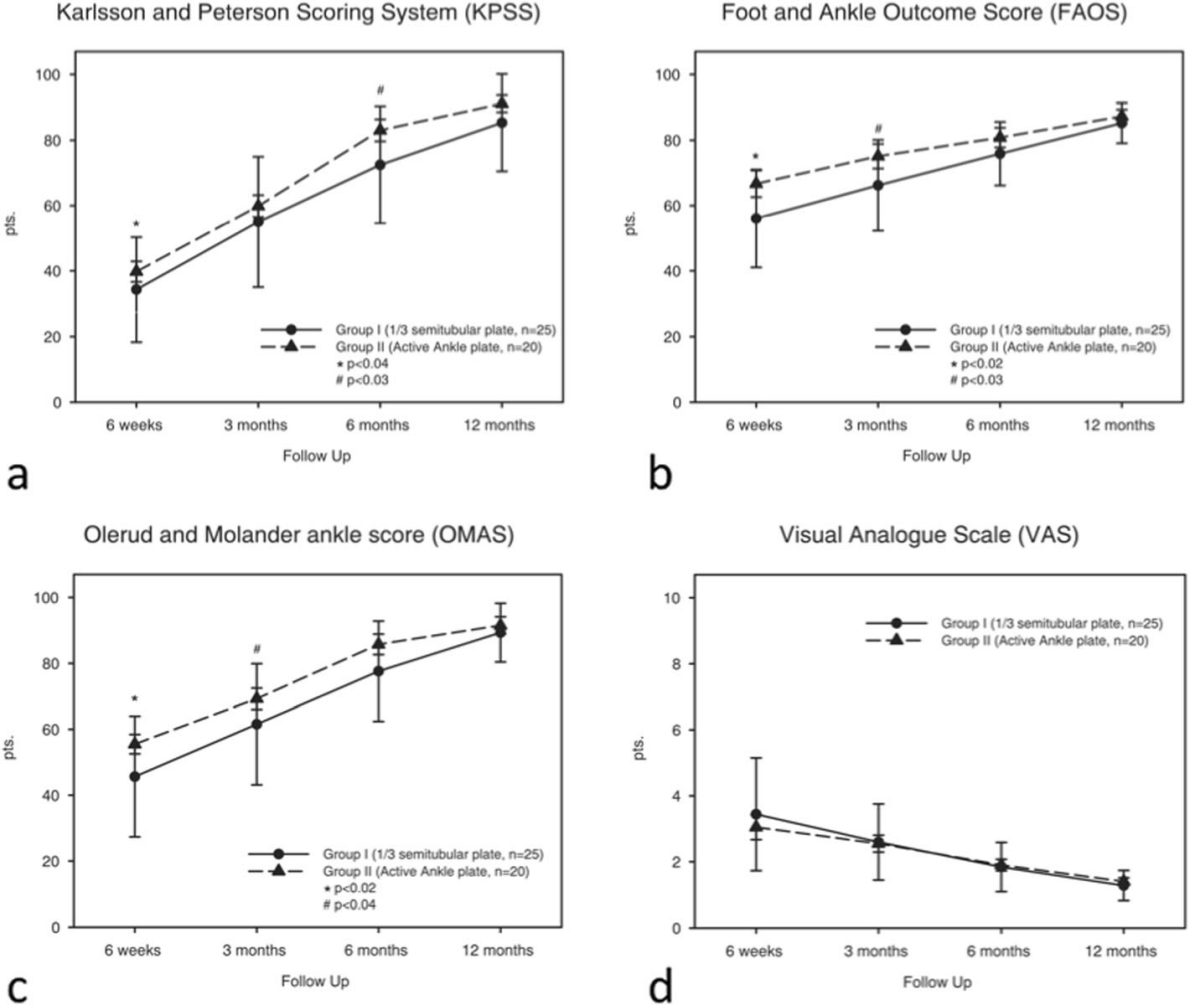
Results of functional Ankle Scores in terms of OMAS, FAOS and KPSS for both groups separately 6 and 12 weeks as well as 6 and 12 months postoperatively. Results are given as ⌀ mean and +/− standard deviation

The first two clinical follow-up exams (after 6 and 12 weeks) showed significant (6 weeks *p* = 0.02, 12 weeks *p* = 0.04) better OMAS results in group II (6 weeks: ⌀56.05 +/− 12, 12 weeks: ⌀69.47+/− 14) compared to group I (6 weeks: ⌀45.22+/− 18, 12 weeks ⌀59.79+/− 16, see Table 4). Similar results for the first two clinical follow-ups were recognized in group II regarding the functional outcome scores FAOS (6 weeks: ⌀66.7 +/− 17, 12 weeks⌀: 75.1 +/− 16, *p* = 0.02, *p* = 0.03) and KPSS confirming the good results of the OMAS. In the later follow-up exams after 6 and 12 months the statistical difference between the two study groups was not statistically significant. The assessed VAS score showed no statistical difference during the entire treatment- well as post-treatment period (see Fig. 1).

In 23 patients (51.1%) an elective hardware removal was performed due to inadvertent or disturbing implant material. In group II the implanted polyaxial locking plate was removed 13 times (56.5%), whereas only 10 patients treated with one-third semitubular plate (group I) requested a removal (43.5%). One plate removal in group II was performed due to a deep wound infection considered as major complication.

No statistical difference between both patient-groups resulted for the ROM in the follow-up-exams (see Table 5).

**Table 5:** Range of Motion at all follow – up dates. Results are given as ⌀ mean and +/− standard deviation

ROM	6 week	12 week	6 month	12 month
**DePuy Synthes®**				
Extension (dorsiflexion)	⌀5 +/−2	⌀ 13+/−5	⌀19 +/−4	⌀22 +/− 2
Flexion	⌀15 +/− 4	⌀25 +/− 4	⌀34 +/− 4	⌀38 +/− 3
**Newclip Technics®**				
Extension (dorsiflexion)	⌀8 +/− 3	⌀16 +/−4	⌀20 +/−4	⌀22+/− 3
Flexion	⌀17 +/− 4	⌀30 +/− 3	⌀35 +/− 4	⌀39 +/− 2

### Radiological follow up

**Fig. 2:**
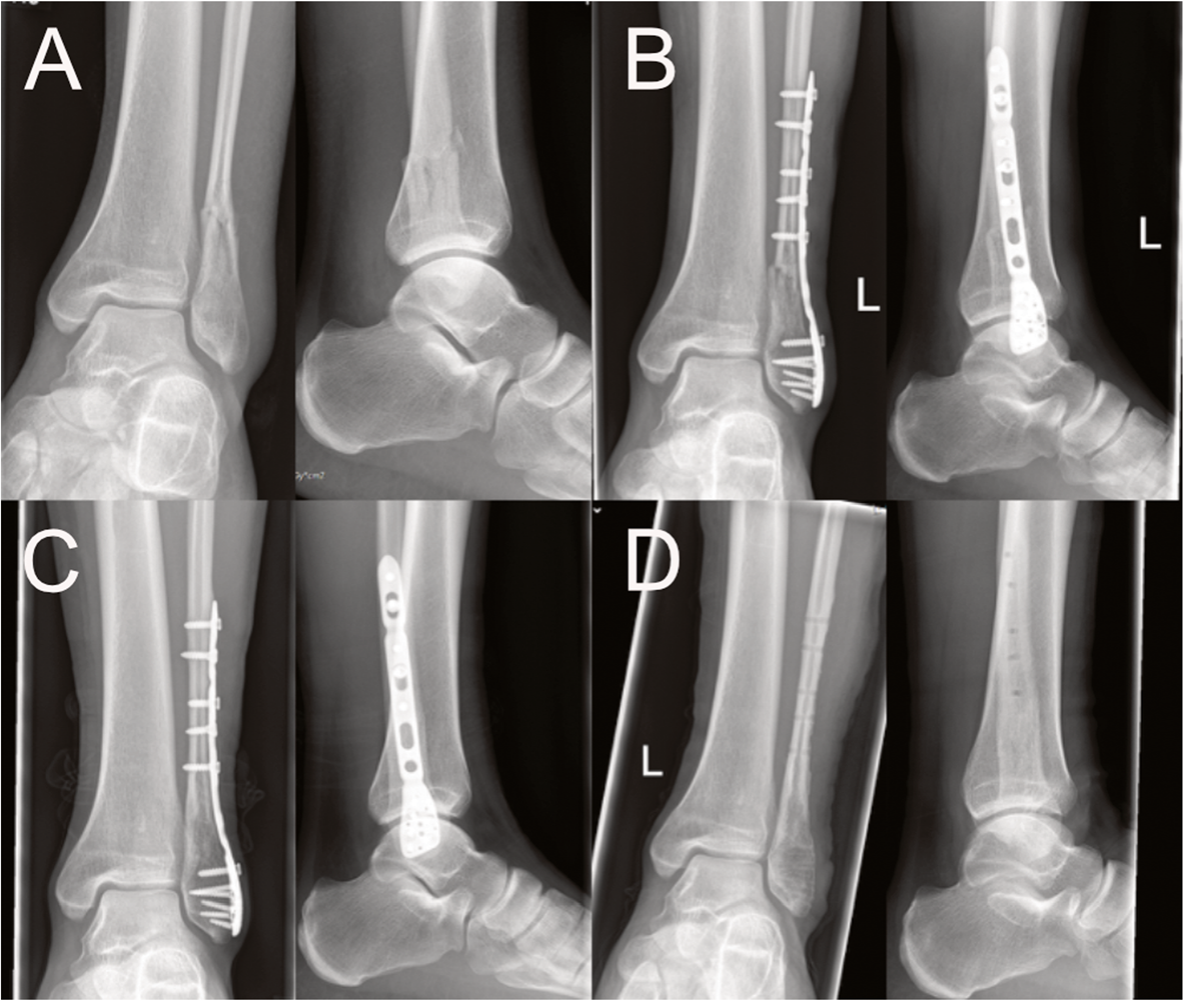
Radiograph of an 18-year-old male patient suffering from an AO type 44B1.1 fracture in two planes (**a**). The patient was treated with the Newclip Technics® Active ankle system. Figure **b** shows the radiographic follow-up 6 weeks after surgery with image **c**) presenting the follow-up after 12 months. Finally image **d**) demonstrates the status after hardware removal (13 months postop)

**Fig. 3:**
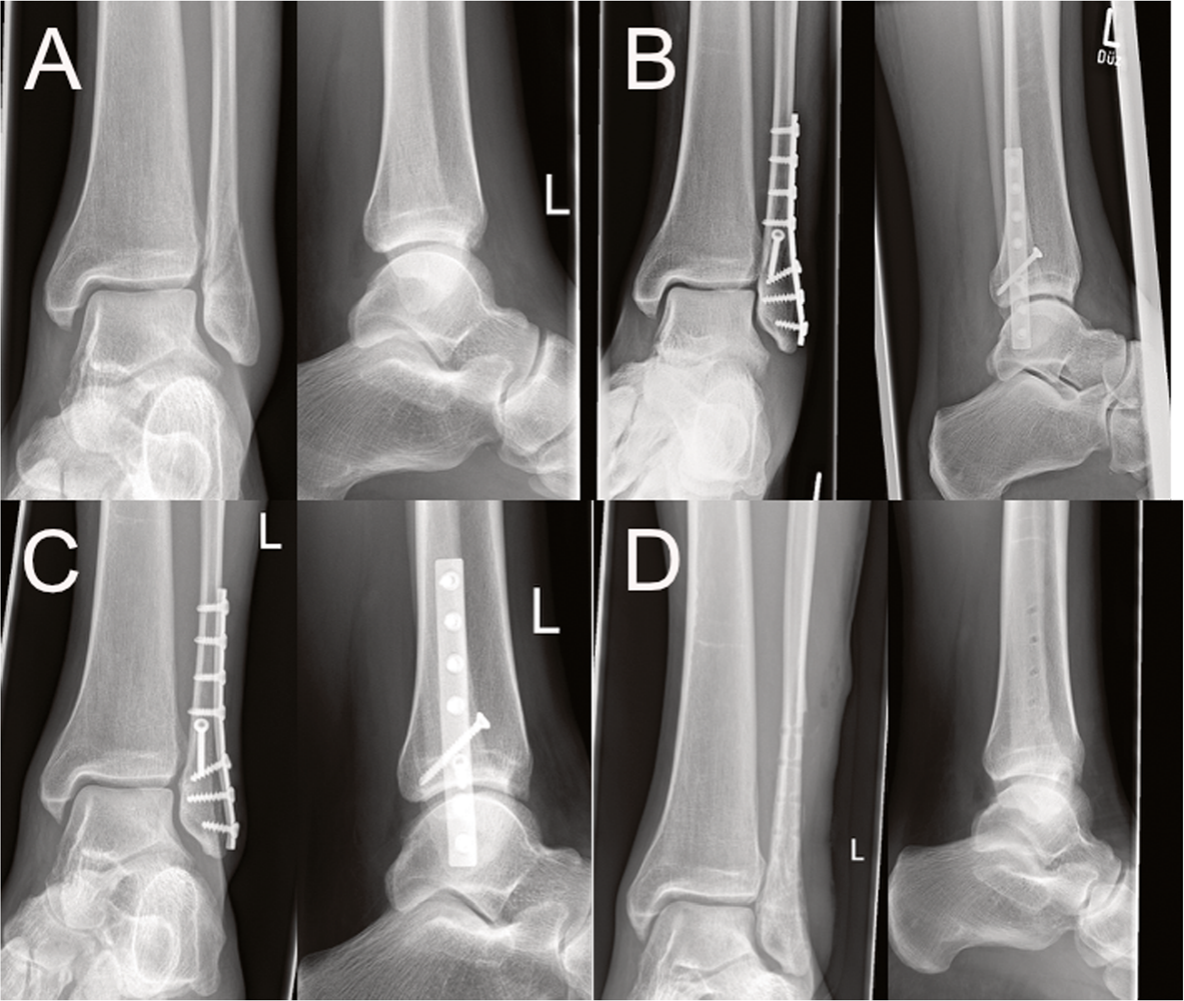
Radiographs in two planes of a 38-year-old male patient with an AO type 44B1.1 fracture (**a**) treated with the DePuy Synthes® one-third semi tubular plate system. Six weeks (**b**) and 12 months (**c**) postoperative follow-up radiographs as well as the control after hardware removal (12 months postop, **d**) are shown as well

The final follow up 12 months after surgery showed complete osseous healing without complications in 44 cases (25 × DePuy Synthes® semitubular plate vs. 19 x Newclip Technics® Active ankle system) (see Figs. 2 and 3). No osseous non-unions were detected. One patient treated by the polyaxial locking plate suffered from a major complication presenting with radiographical signs of screw loosening so that an implant removal was performed. After conversion to conservative treatment radiographical signs of fracture healing without non-union were present after 14 months.

## Discussion

Ancle fractures present a common injury accounting for an incidence of 9% of all fractures of the human skeleton [1]. In the current literature multiple studies have compared conservative and surgical treatment of distal fibula FX [2–4]. In summary open reduction and internal fixation (ORIF) is nowadays considered the standard of care for displaced ankle FX in adults [4–6]. However, to the best of our knowledge there exists no study in the common literature analyzing, whether ORIF of ankle fractures using either a standard semitubular plate or a new polyaxial locking plate system results in a better clinical outcome. Our results demonstrate well that polyaxial locking plates allow for a progressive postoperative rehabilitation protocol with an early start of full weight bearing 3 weeks after surgery leading to reliable results with good functional outcome. A recent advance in the treatment of fractures of different joints was the introduction of anatomically preformed, polyaxial locking plates [29–32, 46]. In addition to their role as internal bone fixator due to their locking fixation, the positioning of the screws can be selected within a total range of up to 30°, allowing for a greater variety of screw orientation with consecutive increased fracture fragment adaptation [30]. So far other studies analyzed the use of polyaxial locking plates in distal fibula FX with regard to osteoporotic bone, complication rates and cost for the health care system [13, 15, 26, 27, 34, 47, 48]. The majority of these studies were of retrospective character or ex-vivo analysis. Therefore the purpose of the presented prospective study was to review the results following treatment with polyaxial locking plate implants in non-osteoporotic patients along with the performance of a progressive postoperative rehabilitation regime.

Biomechanical cadaver studies have shown that modern locking plate systems have significant stronger biomechanical characteristics for distal fibular FX in osteoporotic bone and presented a greater torque compared to conventional plating systems [27, 49, 50].

Aware of these findings along with the fact that early postoperative weight bearing as well as mobilization is a very controversially discussed topic in the recent literature [19, 24, 51–53] the presented results provide a good strategy for a progressive rehabilitation scheme including early weight bearing after a distinct wound healing period. In our opinion the chosen three-week postoperative time interval considered for wound recovery was the key to avoid minor as well as major complications since the distal fibula’s anatomical position is in the subcutaneous tissue without any overlying muscle. In this context wound problems, skin irritation by the implant, metallosis, superficial infections and delayed wound healing are common postoperative complications after ORIF of distal fibula FX [13, 15, 26, 54].

In general delayed wound healing and high complication rates up to 25% were described for ankle FX following surgical treatment [14]. Schepers et al. do not recommend the treatment of distal fibular fractures with semitubular plates since the authors describe an elevated complication rate of 17.5% [15]. In contrast Petruccelli et al. [35] published a study presenting only 8.9% (*n* = 4) wound complications in distal fibular FX treated with locking compression plates compared to a treatment with semitubular plates with 6.3% (*n* = 3) complications without statistical difference between both treatment groups. Similar low infection rates were published by Tsukada et al. [12].

The presented complication rates are consistent with the rates described in the current literature [12, 35]. Group II treated by the polyaxial locking plate showed lower postoperative complications, 2 (10%) minor and 1 (5%) major ones in comparison to group I (semitubular plate) presenting 4 minor (16%) and 2 major complications (8%). However, due to the relatively small study population no significant difference between the two groups was found in this context. Despite the lower complication rate after treatment with polyaxial locking plates, the hereby treated patients required more often a hardware removal. This may be related to a greater implant size due to its slightly bulkier profile.

Substantial differences between both evaluated plating systems were detected in the subjective evaluation of the outcome scores. The used outcome scores are self-assessment questionnaires and are reflecting the subjective physical well-being and clinical outcome of the individual patient [38]. All three outcome scores feature a graduation consisting of five scales ranging from poor, fair, good to excellent. Especially in the early stages of the postoperative treatment, after 6 and 12 weeks, all three applied scores showed significantly different results ranging between fair and good in OMAS, KPSS and FAOS for both study groups. No statistical differences regarding the used outcome scores (OMAS, FAOS and KPSS) were detected in the follow up examinations after 6 and 12 months [13, 55].

These reliable results in the early stages of the postoperative treatment are similar and even better than the results published by Dehghan et al. In their study unstable ankle Fx were treated by ORIF followed by a comparison of 2 different rehabilitation protocols: the one group was allowed for early weightbearing after 2 weeks postoperatively whereas the second group had to follow non-weightbearing for 6 weeks and immobilization. The assessed OMAS after 6 weeks was significantly better in the early weightbearing group compared to the non-weightbearing group (⌀45 vs. ⌀32, *p* = 0.0007) [56]. These results support the hypothesis that early mobilization leads to better clinical outcomes.

Regarding the FAOS, our study shows excellent results in the clinical control after 12 months comparable to those described by Shih et al. [57].

Due to the limited number of enrolled patients the influence of implant removal was not analyzed. However, the presented follow-up rate of 86.5%, the wide assessment of functional parameters and the prospective randomized character certainly present the strengths of the present survey. To the best of our knowledge, moreover, this is the first randomized controlled trial comparing semitubular plates with a polyaxial locking plating system in distal fibula FX treatment applying a progressive early weight bearing regime for the polyaxial locking plating treated patients.

### Limitations

A number of limitations of this study need to be stated. The distinct lack of comparability due to different applied postoperative treatment regimens is considered a basic limitation. While group I - patients (semitubular plate) were treated with partial weight bearing for 6 weeks, group II (locking plate) started full weight bearing after only 3 weeks. However, recommendations for patients treated with locking plates are still missing, the guidelines of the DGOU as well as of the AO for patients treated with semitubular plates involve partial weight bearing [39]. The allowance of early weight bearing lead to improved clinical outcome in patients treated with locking plate systems.

exactly the different postoperative rehabilitation protocol for patients treated with locking plates allowing for an early weight bearing. The presented results seem to be reliable and safe considered as an important contribution of our research.

Besides the small population size only patients treated with standard lateral plate positioning were included, so that patients with posterolateral approach and plate positioning were not considered, presents another study limitation. However, these points should be the scientific focus of future studies.

## Conclusion

The presented study shows that anatomically preformed polyaxial locking plates for the treatment of distal fibular FX lead to good clinical results. Along with an early weight bearing following locking plate osteosynthesis this therapeutical approach seems to represent a safe regimen without secondary loss of reduction. In this context a significant difference in overall clinical outcome between the two used plate systems was observed in the short-term follow-up after 6 and 12 weeks. Although there may be a trend towards the use of locking plate systems in osteoporotic FX in the literature, we demonstrated that this new pre-shaped polyaxial locking plates could bring a benefit to the general population regarding postoperative clinical outcome and shorter time period to full recovery. Overall every single patient might profit from a faster recovery individually but also the cost of the health system will be reduced in general. Further studies with higher sample sizes are necessary to further demonstrate the benefits of these novel implant systems in the treatment of these fractures. In addition, an analysis of the assumed reduction of health related costs is advisable as focus of future studies.

In summary, the successful treatment of distal fibula fractures is essentially dependent on sufficient wound healing and high primary stability for early full weight bearing and initial functional rehabilitation.

## Data Availability

To request the raw data, the first author of the manuscript can be contacted: Michael Zyskowski, Department of Trauma Surgery, Klinikum rechts der Isar, Technical University of Munich, Ismaninger Strasse 22, 81,675 Munich, Germany, Tel.: 0049–89–4140-2126, E-Mail: michael.zyskowski@mri.tum.de

## Abbreviations

DGOU: German Society for Orthopaedics and Trauma
FAOS: Foot and Ankle Outcome Score
Fx: Fracture
KPSS: Karlsson and Peterson Scoring System for Ankle function
OMAS: Olerud-Molander Ankle Score
ORIF: Open reduction internal fixation
